# Metastatic Prostate Ductal Adenocarcinoma Presenting as a Solitary Rectal Mucosal Polyp: A Case Report

**DOI:** 10.7759/cureus.85962

**Published:** 2025-06-13

**Authors:** Lingling Xian, Wei Xin

**Affiliations:** 1 Pathology, University of South Alabama College of Medicine, Mobile, USA

**Keywords:** case report, colorectal metastasis, immunohistochemistry, prostate ductal adenocarcinoma, solitary mucosal polyp

## Abstract

While prostate cancer is predominantly of the acinar subtype, ductal adenocarcinoma is a rare variant that typically presents at an advanced stage, often with low prostate-specific antigen (PSA) levels and rare gastrointestinal metastases. We report the case of a 75-year-old male patient with a history of prostate cancer status post-prostatectomy who, during routine surveillance, exhibited a solitary rectal mucosal polyp. Imaging and colonoscopy identified an invasive carcinoma, mimicking rectal adenocarcinoma, but histologically and immunohistochemically consistent with metastatic prostate ductal adenocarcinoma, isolated to the rectum without other site involvement. The metastatic tumor was surgically removed initially, but local recurrence occurred within a year, and the recurrent tumor was treated with radiation therapy due to inoperability. This case highlights the need to consider metastatic tumor in a patient with a previous history in the differential diagnosis of solitary colorectal lesions and underscores the role of immunohistochemistry in accurate diagnosis and management.

## Introduction

Prostate cancer is predominantly characterized by the acinar type, constituting more than 95% of cases, while ductal adenocarcinoma represents a rare variant, accounting for less than 5% [1]. Ductal adenocarcinoma is known for its propensity to present at an advanced stage with lower PSA levels.

The conventional metastatic spread of prostate adenocarcinoma primarily involves the bone (88.4%), lymph nodes (61.6%), lung (18.7%), and liver (8%), often presenting with multifocal involvement at the time of diagnosis [2]. Gastrointestinal metastasis of prostate cancer is uncommon [2,3]. While locally advanced prostate tumors may invade rectal tissue, they typically present as an infiltrative mass with an identifiable primary, rather than as an isolated lesion [3]. 

This report presents a case of an elderly male patient with a history of prostate cancer status post-prostatectomy who, during routine surveillance, exhibited an unprecedented metastatic manifestation in the form of an asymptomatic solitary rectal mucosal polyp. This case is distinctive in that it concerns an isolated solitary metastatic tumor in the mucosa and submucosa of the rectum without evidence of metastasis to lymph nodes, bones, or any other location. This case highlights the unusual presentation of metastatic prostate ductal adenocarcinoma and its potential to mimic primary colorectal malignancies. By presenting detailed clinical, radiological, and histopathological findings, we aim to contribute to the existing literature on this rare phenomenon. Additionally, we emphasize the significance of considering prostate cancer and any other potential metastatic tumors in the differential diagnosis of solitary colorectal lesions, especially in patients with a history of tumors.

## Case presentation

**Figure 1:**
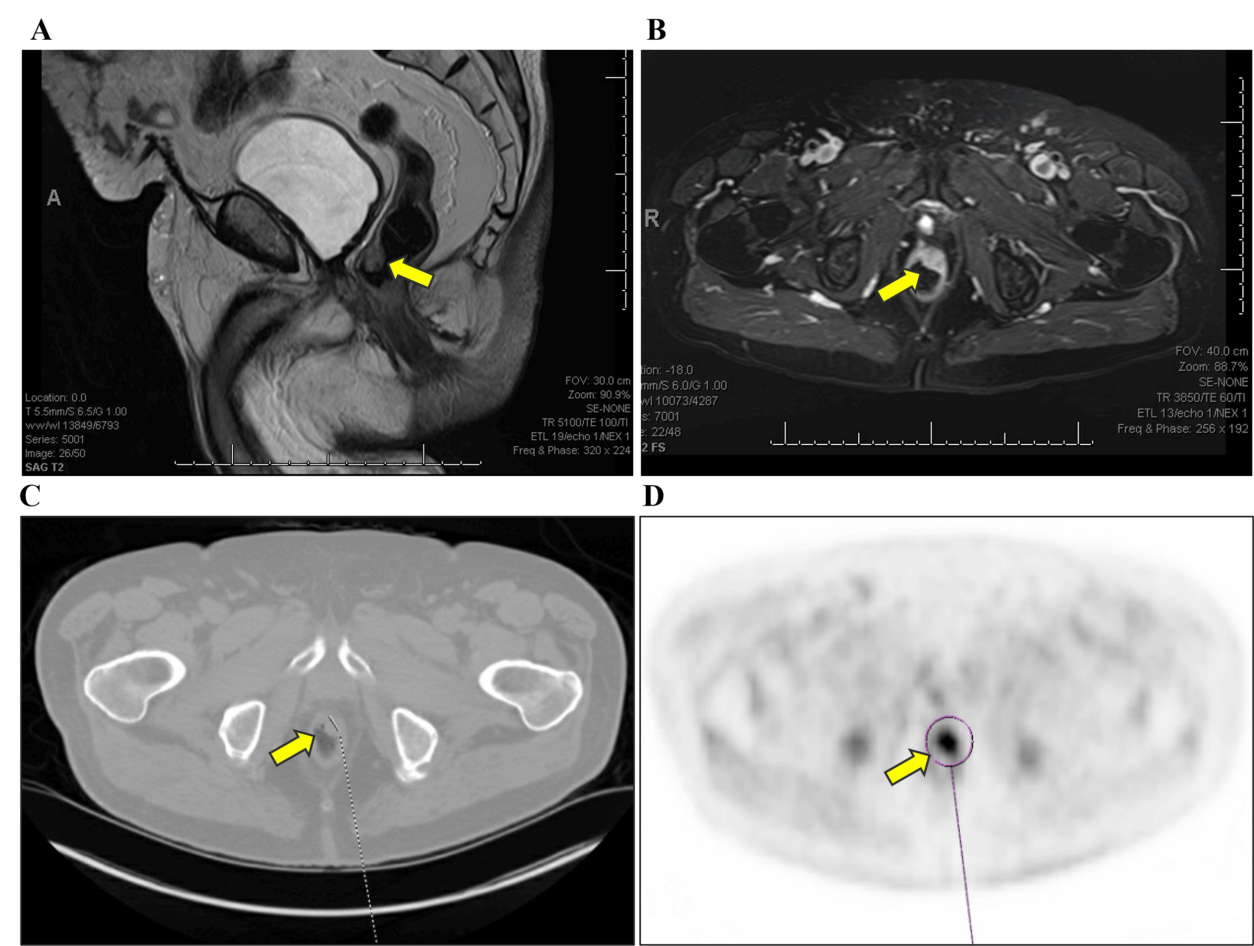
Imaging studies of the rectal polypoid lesion. A and B: MRI showing a solitary polypoid lesion; C and D: PET scan showing a hypermetabolic soft tissue mass in the anterior left rectal wall.

A 75-year-old White male patient, with a medical history significant for hypertension and prostate cancer status post-prostatectomy in 2016, presented to the colorectal surgery clinic following referral by the oncology team. The referral was prompted by an elevated prostate-specific antigen (PSA) of 0.6 ng/ml detected during routine prostate cancer surveillance in September 2021. Subsequent PET scan revealed a hypermetabolic soft tissue mass in the anterior left rectal wall, and an MRI scan showed the polypoid lesion (Figure 1). The PET scan also indicated no bone or any other organ metastases.

A colonoscopy was performed in November 2021, which revealed a suspicious polyp in the rectum. Due to its malignant potential, a biopsy was conducted. Pathological examination of the biopsy indicated high-grade invasive carcinoma, most consistent with prostatic origin, as evidenced by positive immunohistochemical staining for homeobox protein Nkx-3.1 (NKX3.1), cytokeratin AE1/AE3, and cytokeratin 7 (CK7). The polyp was negative for caudal-type homeobox 2 (CDX2), cytokeratin 20 (CK20), p16, p63, and SRY-related HMG-box 10 (SOX10) (data not shown). In contrast, typical colorectal adenocarcinomas are usually positive for CDX2 and CK20, and negative for cytokeratin 7 (CK7), supporting a non-colorectal origin in this case.

Since the polyp was about 3.0 cm in size and could not be completely excised during the colonoscopy, the patient was transferred to our hospital for further evaluation. An MRI of the pelvis in December 2021 revealed an enhancing mass in the proximal rectum, consistent with malignancy, but without signs of locally advanced disease, resembling rectal primary rather than metastatic disease. The patient reported no changes in bowel habits, hematochezia, or alterations in urinary frequency, hesitancy, or stream. Subsequently, the patient underwent a full-thickness excision of the mass via transanal minimally invasive surgery (TAMIS) in January 2022, with all surgical margins reported as negative.

**Figure 2:**
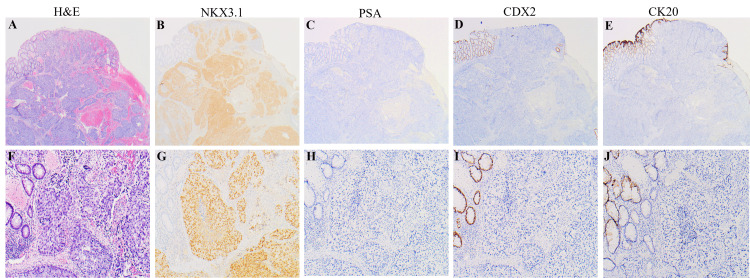
H&E and IHC staining of the metastatic prostate adenocarcinoma. A-E: Low power images (20x), F-J: High power images (200x). A and F: H&E staining; B-J: IHC images of tumor markers; B and G: Homeobox protein Nkx-3.1(NKX3.1); C and H: prostate-specific antigen (PSA); D and I: Caudal type homeobox 2 (CDX2); E and J: Cytokeratin 20 (CK20) IHC: Immunohistochemistry; H&E: hematoxylin and eosin

Histopathological examination of the resected specimen depicted a unifocal, well-circumscribed tumor with large glands composed of tall columnar cells. The tumor showed solid, cribriform, and papillary patterns. There was no colorectal adenocarcinoma precursor lesion, such as tubular adenoma or high-grade dysplasia, identified. Immunohistochemistry showed positivity for NKX3.1, CK7, Cytokeratin AE1/AE3, and negativity for PSA, CDX2, CK20, Thyroid transcription factor 1 (TTF1), P40, and synaptophysin (IHC images are shown in Figure 2), consistent with metastatic ductal adenocarcinoma of the prostate. This metastasis was confined to the rectum only, with no evidence of lymph node involvement, and other common metastatic targeting organs, different from the conventional pattern of prostate cancer dissemination.

Follow-up on the case after the TAMIS treatment

The patient underwent a PET scan in June 2023 due to a sudden increase in PSA levels from 0.2 ng/ml to 0.9 ng/ml within a month. The scan revealed a 3.1 cm soft tissue density mass along the anterior lateral margin of the rectum, showing focal radiotracer uptake consistent with local tumor recurrence. The patient was then evaluated by both a hematologist-oncologist and a radiation oncologist, who concurred that the recurrent mass was inoperable and would be best treated with radiation therapy. Surgical consultation also supported radiation therapy as the definitive treatment due to the firm and fixed nature of the mass. Consequently, the patient was scheduled to begin a course of radiation therapy to target the recurrent tumor.

## Discussion

Ductal adenocarcinoma of the prostate is a subtype of adenocarcinoma that has also been termed endometrioid, endometrial, papillary, or papillary ductal adenocarcinoma [4-6], and papillary or cribriform structures are predominant [7,8]. The hallmark of ductal adenocarcinoma is the presence of tall, pseudostratified columnar epithelial cells that resemble those found in endometrial carcinoma. Central comedo necrosis is often observed. PSA levels can be elevated in prostate ductal carcinoma, but they are not always as significant as in acinar adenocarcinoma. In this case, the PSA immunohistochemistry showed negative results in metastatic tumor cells, which may lead to misdiagnosis. Studies have demonstrated that NKX3.1 is a highly sensitive and specific marker for prostatic origin in metastatic adenocarcinomas, making it particularly important for accurate diagnosis in atypical presentations [9,10]. Generally, ductal adenocarcinoma has a poorer prognosis compared to the more common acinar adenocarcinoma due to its aggressive nature and higher likelihood of metastasis [11]. Ductal adenocarcinoma of the prostate metastasises more often to visceral sites that are less commonly seen for acinar type of prostate cancer such as lung, brain, testis, and penis [8]. The clinical course post resection also underscores the aggressive nature of ductal adenocarcinoma. Despite an initial successful resection via TAMIS, the patient in this case experienced a recurrence within a year, demonstrating the tumor's aggressive nature and the challenges in long-term management. The decision to pursue radiation therapy was guided by the tumor's inoperability due to its firm and fixed nature, emphasizing the complexities in therapeutic planning for recurrent or advanced disease [12]. Additionally, the rapid recurrence post-surgery highlights the need for close monitoring and possibly adjunctive treatments in such aggressive cases.

Ductal adenocarcinomas of the prostate may lose expression of typical prostate markers such as PSA, PSAP, and NKX3.1, particularly in advanced or metastatic stages [13,14]. In this case, we observe a slightly elevated serum PSA level during routine surveillance, while tissue IHC for PSA is negative. The potential explanations may account for this discrepancy include: (i) The tumor may still produce PSA at levels sufficient to be detected in the serum, but below the threshold for IHC detection in tissue sections, and (ii) There may be a minor component of acinar-type adenocarcinoma mixed with ductal carcinoma [1,4]. Acinar components can secrete PSA, whereas ductal elements often lack PSA expression.

The rarity of this case lies in the unusual metastatic site, presentation, and the less common subtype of prostate adenocarcinoma. Prostate cancer metastasizing to the colon as a solitary mucosal or submucosal polyp is exceedingly uncommon. Most documented cases of colorectal metastasis present with multiple lesions or diffuse infiltration rather than a solitary mucosal polyp [15]. Rectal involvement can occur via three distinct pathways: direct invasion through Denonvilliers' fascia and rectal infiltration, lymphatic spread through the common pelvic lymph node channels, and implantation along a needle biopsy tract in rectal or perirectal tissue [3,16]. In this patient, no lymph node metastasis or directly infiltrative invasion pattern was detected, suggesting that the solitary mucosal lesion could be due to previous needle biopsy implantation.

The metastatic prostate adenocarcinoma manifested as a solitary lesion involving mucosa and submucosa in the colon, closely resembling primary colorectal cancer [17]. This atypical presentation underscores the importance of considering prostate cancer in the differential diagnosis of rectal polyps, especially in patients with a history of prostate cancer. Early recognition of such cases can significantly impact treatment decisions and patient outcomes. The key diagnostic pearls for these rare cases include: (i) Colorectal mucosal polyp should never be assumed to be the primary colorectal lesion; (ii) A broad differential should be kept in elderly male patients with a history of prostate cancer, especially with odd polyp histology with no colorectal precursor lesions; (iii) Ductal adenocarcinoma of the prostate may lack PSA positivity [1,4]; therefore, when PSA or PSAP are negative on IHC staining, additional prostate cancer markers should be assessed to avoid missing the diagnosis; and (iv) NKX3.1 is a more reliable prostate marker, especially in poorly differentiated or ductal variants [9,10].

The current report re-evaluates traditional views on metastatic spread, highlighting the necessity for clinicians to consider metastatic prostate cancer in patients presenting with atypical solitary colorectal lesions, especially those with a history of prostate cancer. The immunohistochemical profile is crucial in differentiating primary colorectal malignancies from metastatic prostate cancer, with markers such as NKX3.1 and PSA aiding in accurate diagnosis despite the latter's absence in this case [18]. 

This report adds to the limited literature on solitary colorectal metastasis from prostate ductal adenocarcinoma, emphasizing the need for vigilance in clinical practice. Awareness of these unconventional presentations is crucial for accurate diagnosis and tailored therapeutic strategies. Our findings contribute to the expanding spectrum of prostate cancer metastasis patterns, facilitating better comprehension and management of this rare entity.

## Conclusions

This case underscores the importance of considering metastatic cancer in the differential diagnosis of solitary colorectal polyps, particularly in patients with a previous history of cancer. It also highlights the diverse metastatic potential of prostate ductal adenocarcinoma and the pivotal role of IHC in accurate diagnosis, particularly emphasizing the critical role of NKX3.1 as a more reliable marker in atypical presentations. Increased awareness and vigilance among clinicians can lead to timely and appropriate management of such atypical presentations, potentially improving patient outcomes.
